# A Predictive Spatial Distribution Framework for Filovirus-Infected Bats

**DOI:** 10.1038/s41598-018-26074-4

**Published:** 2018-05-22

**Authors:** Graziano Fiorillo, Paolo Bocchini, Javier Buceta

**Affiliations:** 1 Department of Civil and Environmental Engineering, ATLSS Engineering Research Center, Lehigh University, Bethlehem, PA USA; 2 Department of Chemical and Biomolecular Engineering, Lehigh University, Bethlehem, PA USA; 3 Department of Bioengineering, Lehigh University, Bethlehem, PA USA

## Abstract

Tools with predictive capabilities in regards of filovirus outbreaks are mainly anthropocentric and have disregarded the ecological dimension of the problem. Here we contribute to shift the current paradigm by studying the dynamics of the putative main zoonotic niche of filoviruses, bats, and its link to environmental drivers. We propose a framework that combines data analysis, modeling, and the evaluation of sources of variability. We implement a regression analysis using factual data to correlate environmental parameters and the presence of bats to find the distribution of resources. The information inferred by the regression is fed into a compartmental model that describes the infection state. We also account for the lack of knowledge of some parameters using a sampling/averaging technique. As a result we estimate the spatio-temporal densities of bats. Importantly, we show that our approach is able to predict where and when an outbreak is likely to appear when tested against recent epidemic data in the context of Ebola. Our framework highlights the importance of considering the feedback between the ecology and the environment in zoonotic models and sheds light on the mechanisms to propagate filoviruses geographically. We expect that our methodology can help to design prevention policies and be used as a predictive tool in the context of zoonotic diseases associated to filoviruses.

## Introduction

A large percentage of emerging infectious diseases affecting humans have an animal origin: zoonoses. In particular the *Filoviridae* family causes severe hemorrhagic fevers, e.g. Ebola, in human and nonhuman primates. Ebola is indeed among the zoonotic diseases with highest mortality rate, up to 90%^1^: the 2014 epidemic in West Africa has been the largest registered ever, with around 28,000 probable human cases and a 69% fatality rate^2^. Therefore, understanding the factors and the mechanisms that lead to outbreaks associated with filoviruses is extremely important.

The Ebolavirus (EBOV) strain found during the 2014 outbreak in West Africa was identified as Zaire’s^3^. This suggests the importance of mobility factors of wildlife vectors in zoonoses^4^, even if there are no definitive proofs on the reservoir origin of the outbreak. The World Heath Organization concluded that, prior to the onset, the child that originated the epidemic was seen playing in his backyard near a tree heavily infested with bats and pointed to the exposure of a human to bats as the cause. However, no epidemiologic or genetic data associate this putative reservoir species with the 2014 outbreak^5^. Nonetheless, several other studies have linked filoviruses to bats ecology. Leroy *et al*. reported in a seminal study that anti-EBOV antibodies and EBOV RNA were detected in fruit bat species^6^. Further studies have confirmed this finding^7,8^. In addition, there is evidence of Ebola outbreaks due to a direct exposure of humans to bats in Africa^9^. In this context, the difficulties for identifying the main zoonotic reservoir is partly due to the large diversity of EBOV hosts^1^. In fact, even if bats were indeed the main and unique reservoir of filoviruses as EBOV, most outbreaks might not be primarily driven by direct human-bat contacts, but by other means, such as infected primate carcasses (bushmeat), direct contact with other infected hosts, or contact with bat feces. Recent studies have carefully reexamined the evidence in favor and against bats as the reservoir of filoviruses including EBOV^5,10–12^. Regardless of the controversy in the field and the acknowledgment that Ebola and other filovirus diseases are a multifactorial problem, bats have been identified as an important (direct and/or indirect) driver of outbreaks of these diseases. The fact that bats are able to clear filovirus infections^13^ and travel thousands of kilometers per year in search of better environmental conditions (e.g.,food, shelter)^14,15^ raises the question of how their infection and migration dynamics shape the zoonotic niche of filoviruses. In this study we aim at addressing that question using a novel modeling framework.

During the past years the modeling efforts in the context of filoviruses have been intense^16–20^. In particular, a large number of mathematical models have been proposed to understand the West Africa EBOV infection dynamics from different perspectives, including epidemic, genomic, and social data^16–23^. These models have been shown to provide estimates of the progression of the disease in the short-term (weeks) once an outbreak occurs. However, besides the success of these approaches in describing the dynamics of the infection in human populations, only recently there has been an effort to link the appearance of outbreaks using a zoonotic perspective^24–28^. In this regard, we hypothesize that the infection dynamics of bats can be correlated with filovirus outbreaks. To that end, we introduce here a predictive framework that integrates data and modeling to study the infection dynamics of bats driven by enviroclimatic factors.

Our framework combines a statistical regression of factual data to estimate the dependence of the carrying capacity on environmental parameters, a sampling technique to account for the variability of parameters, and a compartmental model that describes the filovirus infection dynamics in chiroptera species^28^. Notably, when comparing our results with the 2014 Ebola epidemic data, we show that our approach is able to pinpoint geographical locations where, and time points when, EBOV infected bats emerged. This in turn may explain the suggested link between resources in a region and the onset of the Ebola epidemic in West Africa^4^. Therefore, we expect that our methodology can be used to design prevention policies and as an additional resource for reducing the risk of future spillovers.

## Results

### Data Regression Analysis Reveals Key Environmental Parameters in the Ecology of the Zoonotic Niche

In order to clarify what key environmental parameters drive the ecology of the zoonotic niche, we implemented a regression approach to correlate environmental factors relevant to bats ecology with their population at specific locations (see Methods). Environmental/climate data was retrieved using the Google Earth Engine tool to access the databases from NASA Land Processes Distributed Active Archive Center (LP DAAC), USGS/Earth Resources Observation and Science (EROS) Center, Sioux Falls, South Dakota. For data about bat colonies at specific locations we used the data collected by Bergmans^29^. In order to improve the statistics of our approach, we also included expected presence and absence data about bats using the IUCN database^30^. We notice that although there is some data available about bats obtained from serological surveillance studies indicating the infectious state, we did not consider it in our approach to avoid possible artifacts. In fact, as recently pointed out by Leendertz and coworkers, “Bats have longer life spans than most other mammals of similar size, can fly over great distances, and are able to clear filovirus infections. This means that seropositive specimens may be sampled for a relatively long time in areas geographically distant from where the specimens were exposed to ebolaviruses. Hence, the sampling area might not represent the original infection hot spot.”^13^. Consequently, we mostly rely on observations reporting the presence of bats colonies and expected presence/absence and predict the infectious state using our modeling SIR framework (Methods).

In terms of the input, we divided the set of observations (presence-absence data) into training and testing sets using different percentages (training/test): 30%/70%, 50%/50% and 70%/30%. Our results indicated that there was not significant difference in the accuracy of the regression using different training/testing ratios. As for the environmental/geographical parameters, we tested the enhanced vegetation index (EVI), precipitation (PRE), daily air temperature (TMP), land cover index (LND), ground elevation (ELV), human population density (POP), latitude (LAT), and longitude (LON)^31–37^ (see Methods for dimensional treatment). Thus, in addition to the environmental conditions at specific locations, we used variables that account for information related with the vegetation and human settlement since some evidence suggests that forest loss could drive bats (and other possibly infected hosts) into closer contact with humans.

**Figure 1:**
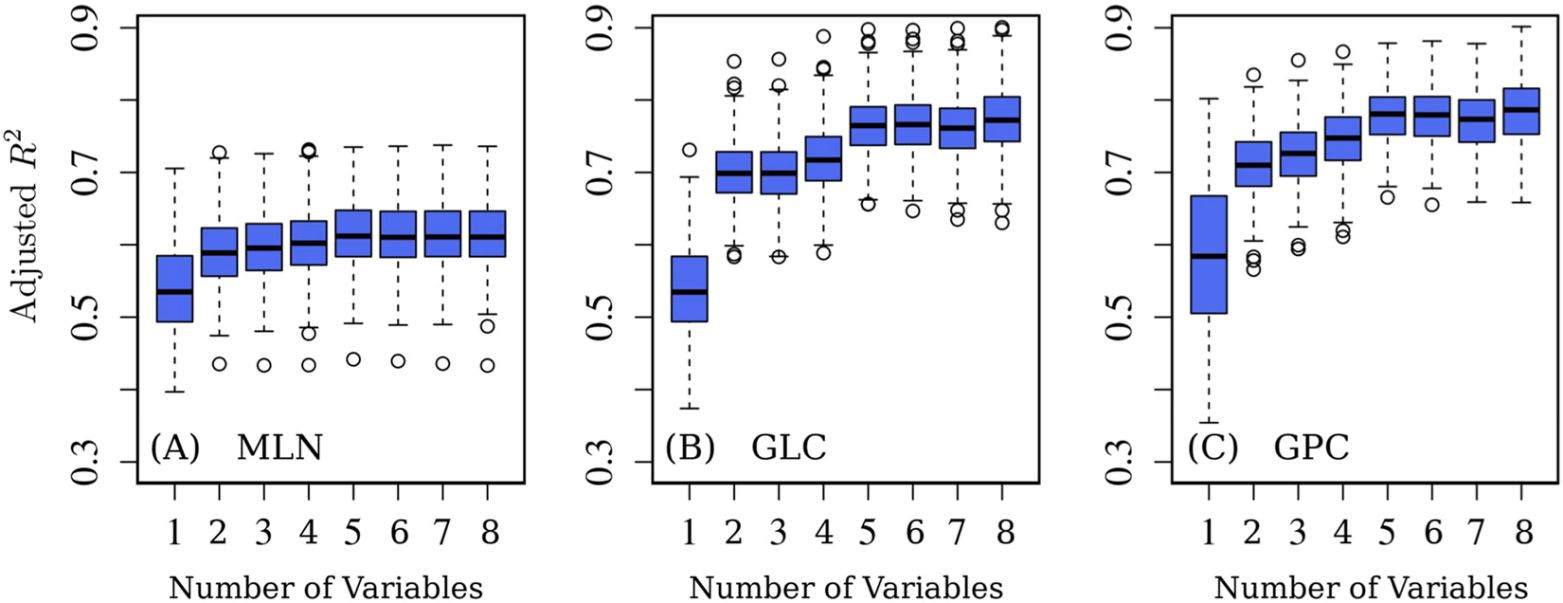
Accuracy of the regression models for *K*_0_: adjusted *R*^2^. In all panels the number of variables from 1 through 8 corresponds to increasing sets of environmental predictors in the following order: {*EVI*, *PRE*, *TMP*, *LND*, *ELV*, *LON*, *POP*, *LAT*}. (**A**) Multi-linear regression (MLN), (**B**) multi-linear regression with linear cross terms (GLC), and (**C**) second order polynomial with linear cross terms (GPC). In each boxplot, the tick mark inside the box indicates the median value of the adjusted *R*^2^, while the vertical extensions of the box above and below the median are the 25^th^ and 75^th^ quartiles, respectively. The height of the box stands for the interquartile range. The whiskers (dashed lines) indicate the spread of points outside the interquartile range. Outliers more than 1.5 times the interquartile range away from the median are indicated by circles. As the number of variables increases, both whiskers and interquartile ranges reduce, indicating that the robustness of the regression improves. The median value of the normalized residual standard error (RSE) for MLN, GLC, and GPC models with five predictors are 35.0%, 27.0%, and 27.0%, while the adjusted *R*^2^ values are 0.62, 0.78, and 0.79, respectively.

The tested variables were sorted by increasing value of the correlation with the observations of bats in the sample and so ranked in the following order, EVI, PRE, TEM, LND, ELV, LON, POP and LAT. We repeated the regression analysis 500 times, splitting the random samples of observations into training and testing sets (training/test ratio 50%/50%). For each random partition of the data in training and testing sets, the least-squares method provides a slightly different estimate of the coefficients *α*’s that weight the relative importance of the variables, (see Methods), which in turn gives slightly different estimates of the carrying capacity *K*_0_ at the various locations. Our results indicate that, as expected, the accuracy of all regression models tested increases as the number of environmental variables increases. Yet, regardless of the regression model, the accuracy reaches a plateau when the following five predictors are considered: EVI, PRE, TMP, LND, and ELV, see Fig. 1. The additional variables (POP, LAT and LON) do not increment significantly the accuracy. This suggests that their contribution is embedded within the other variables.

The accuracy in the calibration of *K*_0_ increases as cross terms between independent variables measuring non-linear relations among variables are added to the model. Figure 1 shows the adjusted *R*^2^ coefficient for the three considered regression models as a function of the data variables by means of a boxplot. The GLC regression model provides a good fitting such that, using just the five predictors mentioned above, the median of the normalized residual standard error (RSE) and adjusted *R*^2^ are 27.0% and 0.78 respectively. While the GPC model provides a similar accuracy, the lack of a significant improvement does not justify the use of a more complex model. We notice that our results show that cross terms are required in the regression and, consequently, the carrying capacity is not simply the result of an additive process of environmental factors but a non-linear combination of them.

In summary, as a function of the relevant environmental parameters, using the GLC regression model, the carrying capacity reads:

1 $$\begin{array}{rcl}{K}_{0}(x,y) & = & 23+736\cdot EV{I}_{n}+283\cdot PR{E}_{n}-95\cdot TM{P}_{n}-281\cdot LN{D}_{n}\\  &  & +\,994\cdot EL{V}_{n}-2203\cdot EV{I}_{n}\times PR{E}_{n}-585\cdot EV{I}_{n}\times TM{P}_{n}\\  &  & +\,191\cdot EV{I}_{n}\times LN{D}_{n}-252\cdot EV{I}_{n}\times EL{V}_{n}+1640\cdot PR{E}_{n}\times TM{P}_{n}\\  &  & +\,-\,185\cdot LN{D}_{n}\times PR{E}_{n}+400\cdot LN{D}_{n}\times TM{P}_{n}-2023\cdot EL{V}_{n}\times TM{P}_{n}\\  &  & +\,277\cdot EL{V}_{n}\times LN{D}_{n}-142\cdot EL{V}_{n}\times PR{E}_{n}\end{array}$$

where the subscript, *n*, indicates normalized dimensionless indexes and the numerical factors are given in units of bats/km^2^ (see Methods).

**Figure 2:**
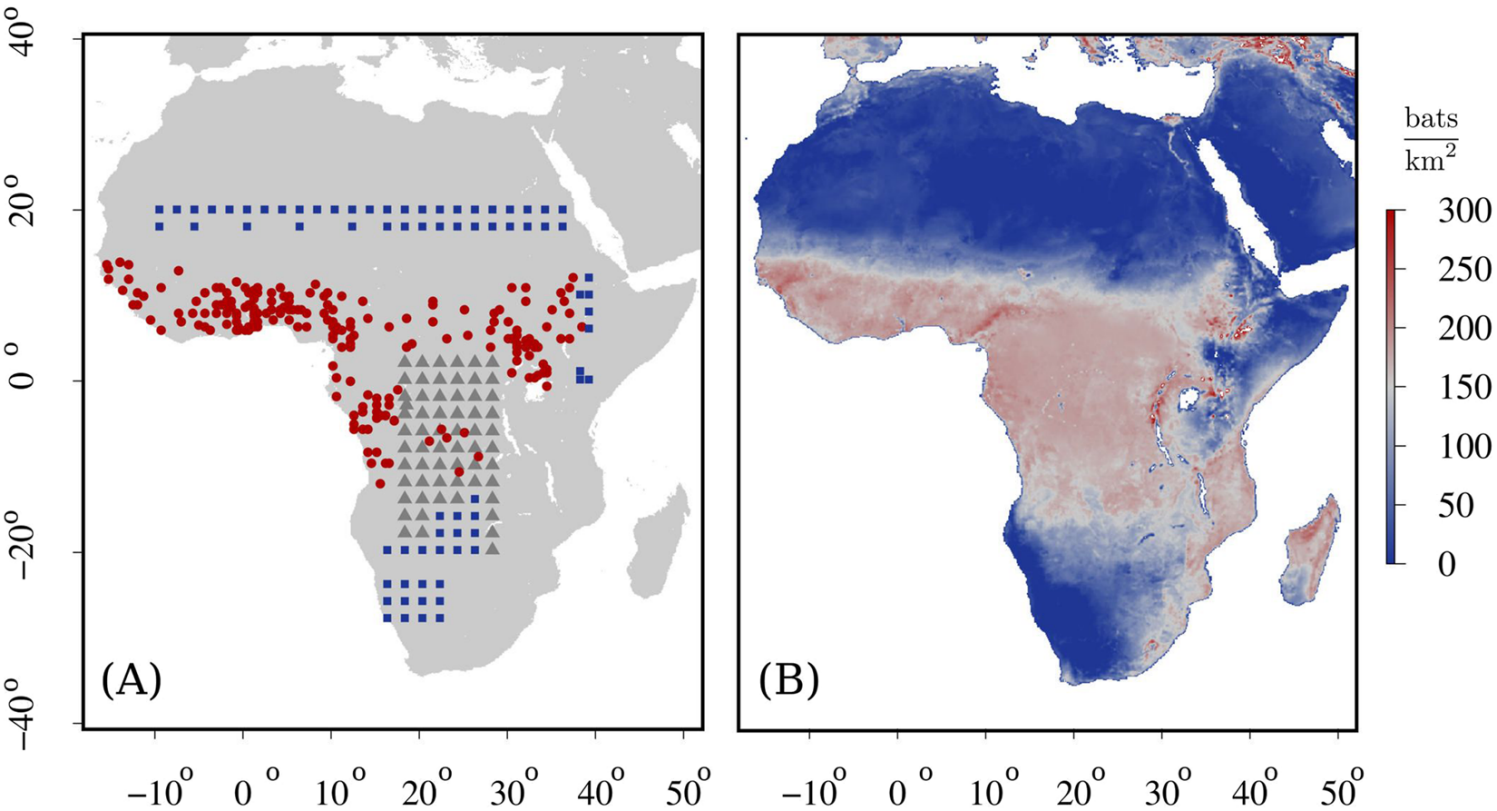
Bat data set and inferred carrying capacity. (**A**) Location of the 334 samples used for the calibration of the regression model (see Methods). Blue squares: *absence* bat data (75 instances); Red circles: *presence* bat data from field studies (198 instances); Gray triangles: show *expected presence* data based on information retrieved in the International Union for Conservation of Nature and Natural Resources (IUCN) database [30] (61 instances). (**B**) Density plot of the calibrated carrying capacity, *K*_0_, as obtained by the generalized multi-linear regression model with linear cross terms (GLC), see Results. *K*_0_ peaks at regions with vegetation levels and temperature range characteristic of the equatorial rainforest and the savanna, and by the Indian Ocean coast of Africa. Map images were generated using R software packages “maps”, “gdal” and “raster”^55–57^.

Figure 2 shows the inferred map for *K*_0_(*x*, *y*) and reveals that the model, Eq. (1), allocates abundant resources in the tropical area of the rainforest and the surrounding savanna, while assigns a scarce value to *K*_0_ in the desertic regions of Sahara and Namibia, as expected. The figure also shows that *K*_0_ is rich in the east area of Africa along the coast of the Indian ocean from Somalia through Mozambique and in the Madagascar island (see Discussion). Notably, some of the considered species are believed not to be present in those geographical locations but we stress that we aim at capturing a generic, effective, behavior of all bats species able to act as a vector of filoviruses. Importantly, this spatial distribution map of *K*_0_ is consistent with the results obtained by other approaches using alternative machine learning techniques^38,39^. Also, the fact that the regression method predicts correctly the presence of bats at locations where no observations where provided, e.g. Madagascar^38,39^, shows the robustness of our approach to obtain a general spatial distribution map of the carrying capacity in the context of bat species associated with filoviruses.

Once the carrying capacity for the bats has been calibrated, we use this information in a SIR mathematical model that estimates the density of bats in different infectious states (see Methods).

### Case Study: Liberia, Sierra Leone, Guinea

In order to test the validity of our framework against epidemic data, we focus on the 2014 Ebola outbreak. Thus, we run simulations of the SIR model using for each month of the year average values of the environmental parameters over 15 years from 2000 to 2015, in the region of Africa where the 2014 outbreak developed. Ideally, we would test our results against bat serosurvey datasets (see^10^) instead of against human cases initiating spillovers. However, the aforementioned lack of precise and recent time stamps of the serosurveys makes it difficult to correlate them with our results. Yet, as shown below, our data agrees with current knowledge about bat infection. Geographically, the first human case was identified in the district of Gueckedou, at the village of Meliandou (Lon: −10.13°, Lat: 8.62°), in Guinea, then the epidemic rapidly spread in Liberia (78 cases) and Sierra Leone (254 cases)^40^.

We focus our analysis on a region centered at Meliandou that comprises 10^6^ km^2^ at a resolution (grid) of 10 × 10 km^2^: the minimum/maximum longitude and latitude range from −17.0° to 7.0° and from 4.0° to 14.0° degrees, respectively. That is, we obtain results for ~10^4^ locations. Our approach additionally provides a monthly temporal resolution for each of those points. To conveniently sample the different parameters of the SIR model (see Methods) we generate 512 realizations using Latin hypercube and compute bounds on the averages by performing a sensitivity analysis on *β* = {0, 0.5, 1.0}, the parameter that weights the importance of the environmental pressure over the bat migration process.

**Figure 3:**
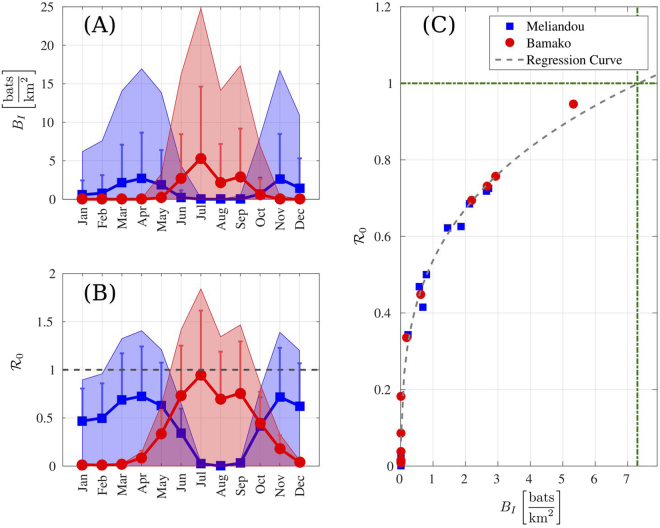
Density of Infected bats and basic reproduction number. In all panels blue/red accounts for Meliandou/Bamako respectively. The time series include one year of predictions based on monthly averaged environmental parameters from 2000 to 2015. The density of infected bats, (**A**), indicates that typical values (for *β* = 0.5) at Meliandou are ≈2bats/km^2^ but environmental factors can increase at times this value to ≈15bats/km^2^, e.g. July at Bamako. The shaded areas show the bounds for the infected bats as a function of *β*. When the environmental pressure pushes K^*^ → *μK*_0_, the infected bats density increases (upper bound of the shaded areas) and reduces when K^*^ → *K*_0_ (lower bound). As expected the basic reproduction number, ${ {\mathcal R} }_{0}$, panel (B) correlates with the density of infected bats as shown in panel (C). The disease propagates among the bats population as long as ${ {\mathcal R} }_{0} > 1$. This requires more than 7.30bats/km^2^ infected bats. This prediction is based on a regression of the correlation curve: ${ {\mathcal R} }_{0}=-\,0.14+0.67\cdot {B}_{I}^{0.26}$ (*R*^2^ = 0.99), with *B*_*I*_ expressed in $\frac{{\rm{bats}}}{{{\rm{km}}}^{2}}$. The two selected locations display a counterphase behavior due to distinct climate conditions for the same period of the year. Within the confidence bounds of our framework (the bar stands for the standard deviation for *β* = 0.5) our model predicts a peak of infection in December at Meliandou when the Ebola epidemic started.

Figure 3 shows time series of the averages of infected bat density and of the basic reproduction number in two locations within our region of interest: Meliandou and a location far away from there, near Bamako in Mali. We can observe a counterphase seasonal variation. Our results reveal the seasonal appearance of infection peaks depending on the location. Remarkably, a peak of infection at Meliandou is noticeable during the months when the outbreak started. The figure also highlights the importance of coupling the seasonal resources with the infection dynamics: locations with no infection might reveal an outburst associated with changes in the environmental factors (see Movie S1). Our analysis predicts two yearly peaks of infection at Meliandou that coincide with the birthing seasons^41^, in agreement with studies about other filoviruses (Marburgvirus)^42^. However, at Bamako our approach only predicts a single peak during the year and suggests the key role played by environmental factors in EBOV propagation. The inferred correlation between the basic reproduction number and the density of infected bats, Fig. 3C, indicates that the infection among bats propagates when densities of infected bats are above $7.30\frac{{\rm{bats}}}{{{\rm{km}}}^{2}}$.

**Figure 4:**
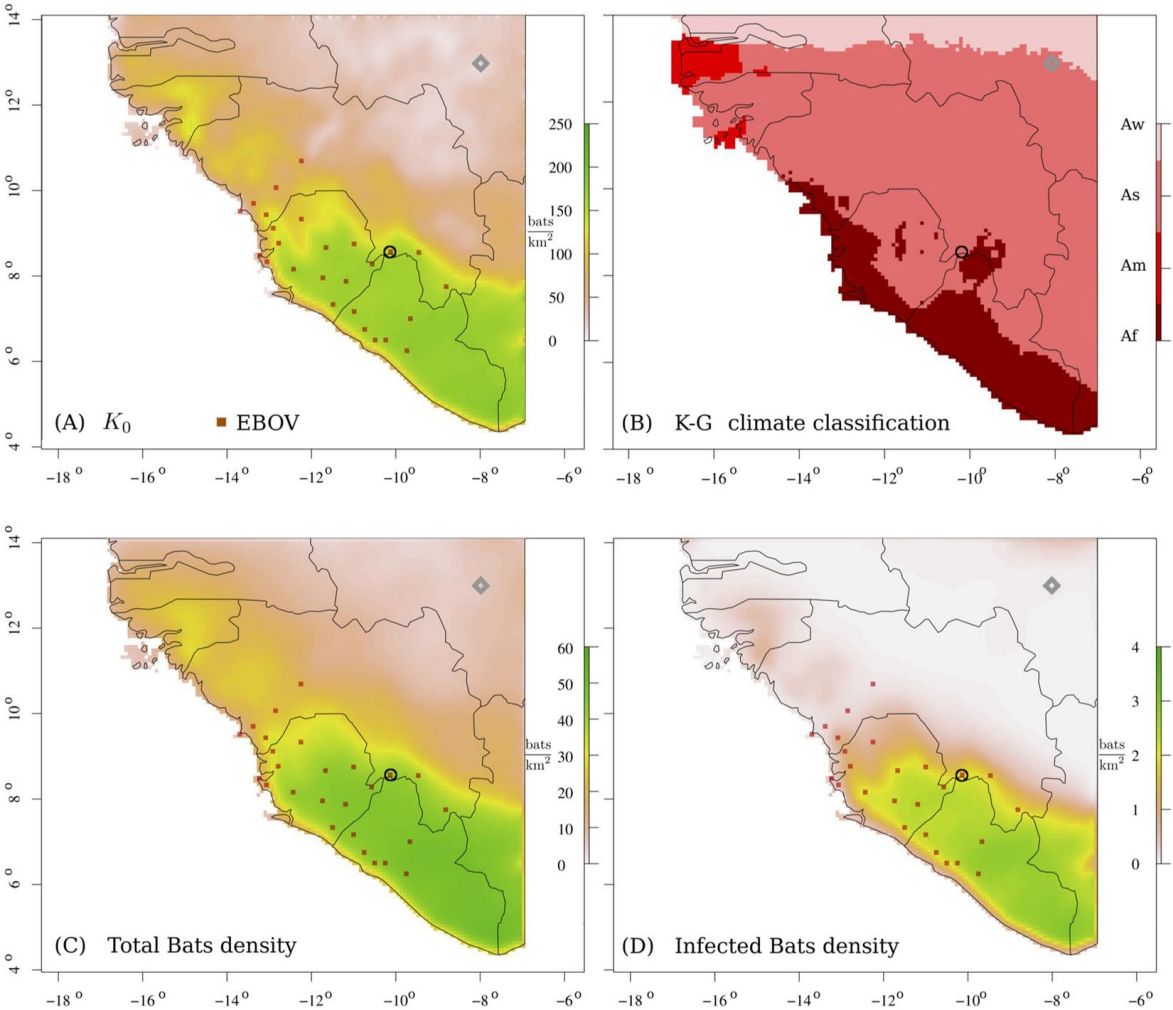
The zoonotic niche and the outbreak of the 2014 Ebola epidemic in West Africa. (**A**,**B**) Inferred bare carrying capacity as obtained by the regression model and Köppen-Geiger climate classification map respectively: *Af*: equatorial rainforest; *Am*: equatorial monsoon (Land cover index 2 in Table 1); *As* and *Aw* are equatorial savannas with dry summer and dry winter, respectively (Land cover index 8 and 9 in Table 1). The confirmed cases of Ebola virus infection in humans in Sierra-Leone, Liberia and Guinea in December 2013 are indicated by means of red squares. The initial location of the outbreak, Meliandou, is highlighted by a black circle. For the sake of comparison, the gray diamond at Lon: −8.00°, Lat: 13.00° pinpoints the location of Bamako where *K*_0_ was low when the outbreak started. (**C**,**D**) Density of total and infected bats respectively. The predicted habitat area for bats extends beyond the region classified as rainforest according to the Köppen-Geiger climate classification^45^ where fruit bats mainly assemble: cf. (**B**,**C**) Map images were generated using R software packages “maps”, “gdal” and “raster”^55–57^.

Figure 4 shows the average estimate of total and infected bat densities obtained with our framework using averages of the environmental data from 2000 to 2015 for the month of December, when the outbreak started (see also Movies S1–S3). On the maps, the location of Meliandou is highlighted with a black circle and the square marks indicate the location of confirmed human cases as collected by WHO^40^.

Our framework is able to link the niche of EBOV infected bats with the region at risk for human infection. Yet, we notice that in the case of the 2014 epidemic only the initial case has been officially linked to a potential contact with an infected animal and genomic data seem to invalidate the possibility of additional instances of human infection due to the zoonotic niche^43,44^. Still, our analysis identifies several risk hotspots for bat infection in the region (Movie S1). In the epidemic region, the estimated percentage of infected bats ranges from 4.0% to 6.0% of the bat population and peaks almost at 8.0% in the South of Liberia and West of Ivory Coast, while in the remaining areas the percentage is below 3.0%. It is also worth noting that this area extends beyond the region classified as rainforest according to the Köppen-Geiger climate classification^45^ where fruit bats mainly assemble and suggests that other bat species play a role in the propagation of the disease.

## Discussion

Here we propose a framework for understanding the spatio-temporal distribution of bats infected by filoviruses that combines data regression, mathematical modeling, and a parameter variability analysis. In this regard, the combination of species distribution modelling with mechanistic epidemiological models is a promising avenue of research for predicting the risk of outbreaks^46^. Here, by training and comparing different regression models, our approach identifies the main environmental parameters that determine the carrying capacity of bats: vegetation index, elevation, precipitation, land cover and air temperature. The inferred carrying capacity is fed into a compartmental SIR model that accounts for the climate/environmental pressure and, together with a sampling technique to account for the parameters uncertainty, we are able to provide quantitative predictions about the dynamics of bat populations in space and time as well as their infectious state.

Our approach is based on a number of assumptions worth discussing. First, in the case of other filoviruses (e.g. Marburg) evidence strongly suggests that bats are the reservoir. Yet, in the case of EBOV, while many researchers support this hypothesis, this issue is still under debate^5,6,10,11,13^. In this regard, our study, by predicting a peak of infectious bats where and when the 2014 outbreak started supports the idea that bats are an EBOV reservoir. Yet, we acknowledge that correlation does not imply causation, and consequently, our study must be considered as an additional, but indirect, argument. In fact, filovirus infection is a convoluted problem where other mechanisms play an all-important role: the effects of other infected hosts, the contact with infected primate carcasses (bushmeat) or bat feces, and/or the human-wildlife interaction are key epidemic drivers that we have disregarded herein. Consequently, our analysis cannot be considered as a way to pinpoint the sufficient conditions for an outbreak to develop through any mechanism, but as a tool to assess the risk of having sufficient conditions from the point of view of bats populations driven by environmental factors. As a matter of fact, there is no point in analyzing the months following the epidemic spillover with our framework, because then the infection dynamics rely mostly on human-to-human interactions, rather than on the behavior of the zoonotic niche^4^. In any case, our approach and results provide important information that can be used by policy-makers, and during decision-making, for preventing and fighting against filovirus outbreaks and in particular Ebola. Thus, at any given location of Africa our framework delivers guidance about the specific periods of the year for which an outbreak could possibly appear due to the bats. For example, Fig. 3 reveals that Meliandou is mostly at risk during March-May and during November-December and, in contrast, Bamako during the period June-September. This dynamics reflects, in agreement with previous results, the importance of birth pulses in the context of filoviruses^42^. We stress that we do not implement explicitly the existence of birth pulses within our framework; instead we couple the abundance of local resources with the growth rate of the population in our mathematical model and the birth pulses develop as an outcome of enviroclimatic factors. Also, our results help to identify geographical hotspots where surveillance can be more effective (Movie S1). In this regard, recent results have analyzed the importance of forest fragmentation in EBOV outbreaks^47^. The predictions of our framework are in agreement with those results (cf. Figs 1b and 4 in^47^) and highlight the importance of linking environmental pressure and bats mobility^28^. Second, we have calibrated the carrying capacity of our model using only some species of bats. Despite this, the results of our approach are representative also of other bat species that have been found positive for EBOV^11^. In fact, there is a large overlap between the regions where these insectivorous bats are endemic and the areas populated by the fruit bats investigated in our work. As a consequence, we have included environmental conditions that favor also the presence of other species. As a proof of this, our prediction for the carrying capacity (Fig. 2B) indicates presence at locations (e.g. Madagascar) where the fruit bats considered in our study are not present, but insectivorous bats are. In other words, by including in our calibration data regions where multiple bat species coexist we are not restricting our analysis to the species mentioned in the manuscript for calibration. We are aware (see Methods) that the species that are a competent reservoir in the context of filoviruses (in particular EBOV) are still under debate. However, in the context of filoviruses prevention it is better to incorporate a false positive than a false negative. For this reason, we decided to include in the analysis also some species for which the debate is still open. Third, with regard to the mathematical SIR model, we have assumed that the transmission mechanism is density- instead of frequency-dependent. Our assumption is based on the same arguments used by other researchers modeling the filovirus infection dynamics in bats^24^: demographic studies have shown that bats usually roost at high densities that are rather independent of the increase of the colony size^48^ and the transmission of filoviruses among them seems to rely on contact with fluids (feces and urine)^10^. Indeed EBOVs have been isolated from bat feces 21 days after infection^49^. These facts point towards a density-dependent transmission modeling approach^50^.

As for the applicability of our methodology to other locations, our framework was calibrated for the African continent, but adapting it to other geographic areas is straightforward, since the environmental parameters are easily accessible for most parts of the world at high resolution and for several time windows. In that regard, we stress that environmental drivers are crucial, yet insufficient, for establishing the zoonotic niche and data analysis must be complemented by modeling efforts to adequately pinpoint regions at geographical risk of infection. Also, the lack of knowledge of some parameter values as well as their variability requires probabilistic sampling, to address their uncertainty.

Our study also suggests, and the 2017 outbreak confirms, that the diseases with a filovirus origin are latent in the African continent and further outbreaks are likely to appear in the future, unless a widespread vaccination campaign is implemented, when it is available. Also, according to our study, the appearance of outbreaks is tightly linked to fluctuations in the environmental conditions that determine strongly both the migration patterns of bats and their infection dynamics. Among the environmental drivers, both elevation and land cover index are slow-changing variables compared to the vegetation index, the air temperature or the rainfall. Consequently, the sooner and the more comprehensive the information about these factors is available, the better the predictive character of our framework for identifying the risk of outbreaks will be. Recent data initiatives, as Google Earth Engine, certainly contribute positively towards that end. The predictive character of our approach also relies on the quality and the quantity of information about the presence of bats at specific locations and on the quantification of several parameters related to the bats physiology and their interactions with the environment, including birth and death rates, the rate of transmission/recovery of/from the disease, and the rate of depletion of natural resources. In that regard, the broader use of remote telemetry and the systematic implementation of quantitative approaches in ecology will help to cover data gaps.

Another point worth discussing is the role played by bat mobility to spread filoviruses. Here we do not aim at determining the strain propagation mechanisms, i.e. a strain could slowly spread from colony to colony from Central Africa or, alternatively, background infection levels have always been prevalent. Still, we envision a reinforcement mechanism where mobility plays a key role. As shown in Fig. 3, basal levels of infection do not imply disease spreading; yet, population levels can locally increase due to migration and seasonality^28^ thus triggering an outbreak according to our study.

A final comment is about possible strategies to overcome additional sources of uncertainty to accurately set confidence bounds for our predictions. Here we have calibrated the carrying capacity of the zoonotic niche training different regression models by using environmental data for a ten year period. While the accuracy of the regression is good, there is certainly a source of variability in the carrying capacity that we have disregarded. Including that randomness depends on the characterization of this “random field” and a rigorous sampling of its realizations to represent accurately its statistical properties. Given the spatial and temporal resolution considered in our framework, that task is computationally unrealistic, even on supercomputing facilities, when using traditional methods, e.g. Monte Carlo. This poses the interesting question of developing computationally efficient and mathematically rigorous methods to account for this unavoidable source of uncertainty in the context of ecology. Work in this direction is in progress.

## Methods

### Carrying Capacity Calibration and Data Sets

Herein, we aim at obtaining a distribution map of the “bare” carrying capacity as a function of environmental variables that accounts for the available resources for bats species associated with filoviruses in Africa. To that end, in this study we analyze the geographical distribution of four species of bats from sub-Saharian Africa: *Eidolon helvum, Hipposideros gigas, Hypsignathus monstrosus*, and *Rousettus aegyptiacus*^30^. These species were selected because a) there is evidence that suggests their relation with filoviruses infection cases, b) they cluster in large colonies and contribute importantly to the chiroptera biomass, and c) quantitative data is available on their presence at specific locations and colony sizes, which we can use in our regression approach. Some researchers have claimed that *Rousettus aegypticus* does not appear to be a competent reservoir in particular for EBOV^51^. However, this claim is contradicted by the findings of Pourrut *et al*. during the Gabon outbreak, where they tested field bats for Marburg and Ebola^52^.

By mixing species with a variety of life characteristics (i.e. insectivorous and frugivorous bats, tree dwelling and cave roosting bats), we expect to capture effectively a collective, non-specific, trait about the carrying capacity of bats. This overall behavior about bat species is further explored by sampling the parameter space of our model as explained below. We acknowledge that by discarding other bat species that have been shown to contribute to the filovirus zoonotic niche, e.g. *Epomops franqueti* and *Myonycteris torquata*^10^, our results can be thought to be biased. However, we stress that the geographical distribution of these species overlaps with that of the species considered in our study and, as shown in the Results section, our results are in good agreement with current knowledge about the zoonotic niche and future field studies would allow to refine our findings easily by implementing the methodology introduced here.

The maximum size of a colony for each of the considered species is approximately 500,000 *(Eidolon helvum)*, 1,000 *(Hipposideros gigas)*, 100 *(Hypsignathus monstrosus)*, and 5,000 *(Rousettus aegyptiacus)* individuals^30^. Although it is difficult to accurately estimate spatial densities of bats colonies^53^, studies conducted on *Eidolon helvum*, the most common fruit bat in Africa, show that colonies forage in regions near the roost within a radius of action that ranges between 10 km and 20 km^14,54^. Here we assume that a radius of twice the average size of the foraging distance, 30 km, provides a realistic upper bound of the colony area of influence: 2,800 km^2^^14^^,^^15,30^. Accordingly, the density of a *Eidolon helvum* colony for example, can be estimated equal to $180\frac{{\rm{bats}}}{{{\rm{km}}}^{2}}$. We take these densities as indirect measurements of the “bare” carrying capacity, *K*_0_, at a specific location to train different regression models (see below); that is, the maximum density of bats that can be sustained by the available resources in a particular region.

We hypothesize that it is possible to correlate *K*_0_ with a set of environmental parameters that are important for the habitat of fruit bats, such as vegetation, precipitation, temperature and ground elevation. To this end, we implement a regression approach testing three models: a multi-linear model (MLN), a generalized multi-linear model with linear cross terms (GLC), and a second order polynomial model with linear cross terms (GPC).

Input data in the regression models are values of environmental parameters averaged over ten years (period from 2000 to 2010) retrieved from the Google Earth Engine database collection at a resolution (grid) 10 × 10 km^2^. Environmental/climate data was obtained from the databases of the NASA Land Processes Distributed Active Archive Center (LP DAAC), USGS/Earth Resources Observation and Science (EROS) Center, Sioux Falls, South Dakota^31–37^. All map images shown in this study were generated using the R software packages “maps”, “gdal” and “raster”^55–57^.

We tested eight environmental parameters: enhanced vegetation index (EVI, dimensionless), precipitation $({\rm{PRE}},{\rm{units}}:\frac{{\rm{mm}}}{{\rm{5}}\cdot {\rm{days}}})$, daily air temperature (TMP, units: °C), land cover index (LND, dimensionless), ground elevation (ELV, units: m), population density (POP, units: individuals/km^2^), latitude (LAT, units: °), and longitude (LON, units: °)^31–37^.

The EVI accounts for the amount of vegetation at a specific location (0 if low and 1 if high) and optimizes the vegetation signal with improved sensitivity in high biomass regions^32^. Fruit bats are present in geographic regions with medium/high vegetation level. The land cover index, LND, follows the MODIS classification scheme and associates an index to different geographic areas: there are 7 major categories and 17 subcategories listed in Table 1^31,37^. We convert all satellite data entries to normalized dimensionless indexes by using the maximum value found for each variable in the analyzed data (see Results): PRE_*MAX*_ = $100\frac{{\rm{mm}}}{5\cdot {\rm{days}}}$, LND_*MAX*_ = 15, ELV_*MAX*_ = 5, 000 m, TMP _*MAX*_ = 50 °C, POP = $400\frac{{\rm{individuals}}}{{{\rm{km}}}^{2}}$, LON_*MAX*_ = 50°, and LAT_*MAX*_ = 40°. We point out that LND is a categorical, rather than a quantitative variable, but the subcategory indexes are sorted by density of vegetation. Thus, for the sake of consistency with the rest of the predictors, we decided to treat the dummy variable associated with LND using rational numbers in the range [0, 1] rather than integers.

**Table 1:** Land cover MODIS classification scheme.

Forests	Woodlands	Grasses	Shrublands
1 Evergreen Needleleaf forest	8 Woody savannas	10 Grasslands	6 Closed shrublands
2 Evergreen Broadleaf forest	9 Savannas		7 Open shrublands
3 Deciduous Needleleaf forest			
4 Deciduous Broadleaf forest			
5 Mixed forest			
**Unvegetated**	**Croplands**	**Inundated**	
0 Water	12 Croplands	11 Permanent wetlands	
13 Urban and built-up	14 Cropland/Natural mosaic		
15 Snow and ice			
16 Barren or sparsely vegetated			

An additional input to the regression models is the estimated bare carrying capacity, *K*_0_, at locations where field studies have revealed the presence of bat colonies. We use data as collected by Bergmans^29^. We reiterate that although there is some data available about bats obtained from serological surveillance studies indicating the infectious state, we did not consider it in our approach to avoid possible artifacts. Consequently, we rely on observations reporting the presence of bats colonies and expected presence/absence and predict the infectious state using our modeling SIR framework.

We complement this information with (a) *expected* presence data by imposing the presence of bat colonies at locations where the IUCN database (a geographical range database)^30^ suggests the existence of the considered species and, (b), with *absence* data, i.e. *K*_0_ = 0, at locations where environmental conditions challenge bat survival (e.g., Sahara dessert), see Fig. 2. Still, since we aim at correlating as precisely as possible environmental factors and the carrying capacity, we keep the number of these additional instances, where presence/absence has not been certified, to a minimum that yields an acceptable accuracy of the regression analysis.

The regression models used in our approach satisfy the following functional forms:

2 $${K}_{0}(x,y)={\alpha }_{0}+\sum _{i=1}^{p}{\alpha }_{i}\cdot {v}_{i}(x,y)({\rm{MLN}})$$

3 $${K}_{0}(x,y)={\alpha }_{0}+\sum _{i=1}^{p}{\alpha }_{i}\cdot {v}_{i}(x,y)+\sum _{i=1}^{p}\sum _{j=i+1}^{p}{\alpha }_{ij}\cdot {v}_{i}(x,y)\cdot {v}_{j}(x,y)({\rm{GLC}})$$

4 $${K}_{0}(x,y)={\alpha }_{0}+\sum _{i=1}^{p}{\alpha }_{i}\cdot {v}_{i}(x,y)+\sum _{i=1}^{p}\sum _{j=i}^{p}{\alpha }_{ij}\cdot {v}_{i}(x,y)\cdot {v}_{j}(x,y)({\rm{GPC}})$$

where *p* represents the number of environmental variables, the terms *α*_0_ (basal value of the carrying capacity), *α*_*i*_, and *α*_*ij*_ are the regression coefficients $({\rm{units}}:\frac{{\rm{bats}}}{{{\rm{km}}}^{2}})$, and *v*_*i*_ and *v*_*j*_ are the dimensionless environmental variables that are functions of the longitude and the latitude (*x* and *y* respectively). The coefficients of the regression model are obtained by applying the least-squares method (see Results).

### Compartmental SIR Model for Filovirus Infection and Parameter Sampling

Agent-based epidemiology modeling allows a high level of detail. As a consequence, more parameters need to be fitted/estimated and these models are computationally expensive. In case multiple scenarios need to be explored for evaluating uncertainties, as we do here, this modeling scheme is therefore not suitable. In contrast, compartmental coarse-grained descriptions require fewer parameters to fit/calibrate and are computationally efficient. Thus, we couple regression data, i.e. the inferred carrying capacity *K*_0_, with a compartmental model that we recently proposed for the dynamics of bat populations in the context of filoviruses infection: we refer the reader to^28^ for the rationale underlying the different processes considered in our modeling, including the inclusion of the recovery-from-infection state. The model accounts for densities of fruit bats that are either Susceptible, *B*_*S*_, Infected, *B*_*I*_, or Recovered, *B*_*R*_, from EBOV infection:

5 $$\frac{\partial {B}_{S}}{\partial t}={b}_{{K}^{\ast }}B-c{B}_{S}-|{a}_{{K}^{\ast }}|\frac{{B}_{S}B}{K}-e{B}_{S}{B}_{I}+f\,{B}_{R}+{D}_{{K}^{\ast }}{\nabla }^{2}{B}_{S}$$

6 $$\frac{\partial {B}_{I}}{\partial t}=-\,c{B}_{I}-|{a}_{{K}^{\ast }}|\frac{{B}_{I}B}{K}+e{B}_{S}{B}_{I}-d{B}_{I}+{D}_{{K}^{\ast }}{\nabla }^{2}{B}_{I}$$

7 $$\frac{\partial {B}_{R}}{\partial t}=-\,c{B}_{R}-|{a}_{{K}^{\ast }}|\frac{{B}_{R}B}{K}+d{B}_{I}-f\,{B}_{R}+{D}_{{K}^{\ast }}{\nabla }^{2}{B}_{R}$$

8 $$\frac{\partial K}{\partial t}=-\,\gamma B+r({K}_{0}-K)$$

Eqs (5–7) describe the dynamic of bats in space and time as a function of the resources (carrying capacity) *K*, where *B* = *B*_*S*_ + *B*_*I*_ + *B*_*R*_ stands for the total density of bats at a given location.

The parameters of the model are the birth rate ${b}_{{K}^{\ast }}$, the death rate *c*, the virus transmission rate *e*, the recovery from infection rate *d*, and the rate at which bats return to a susceptible state, *f*. In addition, the coefficient ${a}_{{K}^{\ast }}={b}_{{K}^{\ast }}-c$ corresponds to the population growth rate, and ${\nabla }^{2}=\frac{{\partial }^{2}}{\partial {x}^{2}}+\frac{{\partial }^{2}}{\partial {x}^{2}}$ to the Laplacian operator that accounts for the spatial mobility of bats; and ${D}_{{K}^{\ast }}$ is the diffusion coefficient. Notice that in the model, based on the available data about filovirus infection in bats, we consider that the vectors are born virus free^42^. It is worth noting that our model assumes a density-dependent transmission mechanism. This is the same approach used in other models based on the bat roosting, demography, and the means of filovirus transmission^24^ (see Discussion).

Eq. (8) describes the dynamics of resources as a function of the bare carrying capacity *K*_0_ estimated by the regression approach, where *r* represents the natural depletion rate of resources and *γ* is the consumption rate of resources by bats. The environmental pressure is implemented in the model by modulating both the birth, ${b}_{{K}^{\ast }}$, and the diffusion rate, ${D}_{{K}^{\ast }}$, as a function of K^*^. This parameter describes a threshold value of the carrying capacity for which there are changes in ${b}_{{K}^{\ast }}$ and ${D}_{{K}^{\ast }}$. On one hand, if *K* is smaller than K^*^, then ${b}_{{K}^{\ast }}=0$ and ${D}_{{K}^{\ast }} > 0$. That is, if the environmental factors challenge bat survival, then bats migrate. On the other hand, if *K* is greater than K^*^, then ${b}_{{K}^{\ast }} > 0$ and ${D}_{{K}^{\ast }}=0$. If the conditions in terms of the available resources are satisfactory for the sustainability of the population, then bats do not migrate (see^28^ for details). We point out that this birth and mobility dynamics recapitulates effectively two important mechanisms for pathogen spreading: birth pulsating dynamics and habitat fragmentation (see Discussion). As a function of the parameters of the model and the bare carrying capacity, *K*_0_, the basic reproduction number, ${ {\mathcal R} }_{0}$, can be estimated^28^:

9 $${ {\mathcal R} }_{0}=\frac{e}{{e}_{c}}-\,\frac{|{b}_{{K}^{\ast }}-c|}{{K}^{\ast }{e}_{c}}$$

We recall that ${ {\mathcal R} }_{0}$ indicates how the disease propagates among bats, such that if ${ {\mathcal R} }_{0} > 1$ the infection spreads in the population and if ${ {\mathcal R} }_{0} < 1$ the infection dies out^58^.

The system of Eqs (5–8) has three different stationary states, $\{{K}^{st},{B}_{S}^{st},{B}_{I}^{st},{B}_{R}^{st}\}$. On one hand, {*K*_0_, 0, 0, 0} corresponds to the trivial null state: no bats, and the resources are at their bare capacity. This state is stable if ${a}_{{K}^{\ast }} < 0$. The second steady state condition corresponds to the infection-free equilibrium {*μK*_0_, *μK*_0_, 0, 0}, where $\mu =\frac{r}{r+\gamma }$. This state is stable if ${a}_{{K}^{\ast }} > 0$ and the infection coefficient *e* is smaller than a critical value ${e}_{c}=\frac{(c+d)}{\mu (1-\beta ){K}_{0}}$, where the coefficient *β* ∈ [0, 1] tunes the value of K^*^ in the interval [*μK*_0_, *K*_0_]: K^*^ = *μK*_0_(1−*β*) + *K*_0_*β*. For example, when *β* = 0.5, then ${K}^{\ast }=\frac{(1+\mu ){K}_{0}}{2}$. The third steady state corresponds to the infection stage, {*μK*_0_, *α*_*S*_*K*_0_, *α*_*I*_*K*_0_, *α*_*R*_*K*_0_}, that is stable if ${a}_{{K}^{\ast }} > 0$ and *e* > *e*_*c*_, where:

10 $${\alpha }_{S}=\mu (1-\beta )\frac{{e}_{c}}{e}$$

11 $${\alpha }_{I}=\frac{\mu (1-\beta )(c+f)}{(c+d+f)}(1-\frac{{e}_{c}}{e})$$

12 $${\alpha }_{R}=\frac{\mu (1-\beta )d}{(c+d+f)}(1-\frac{{e}_{c}}{e})$$

We solve numerically the SIR model by means of an explicit Forward-Time Central-Space (FTCS) finite differences method in a rectangular lattice^59^. Each tile of the lattice represents 100 km^2^, i.e. a spatial resolution of 10 km that is the same of the geographic maps used for the regression analysis. In order to avoid transient effects in our simulations, the initial values for *B*_*S*_, *B*_*I*_, and *B*_*R*_ correspond to the stationary densities as prescribed by Eqs (10–12). The integration time step in our simulations is $\frac{1}{100}$ days. This guarantees the stability of the integration method and ensures that we capture the smallest time scale relevant in the dynamics of the bats population. Finally, the equations are integrated over one month period to ensure that stationary conditions are reached.

**Figure 5:**
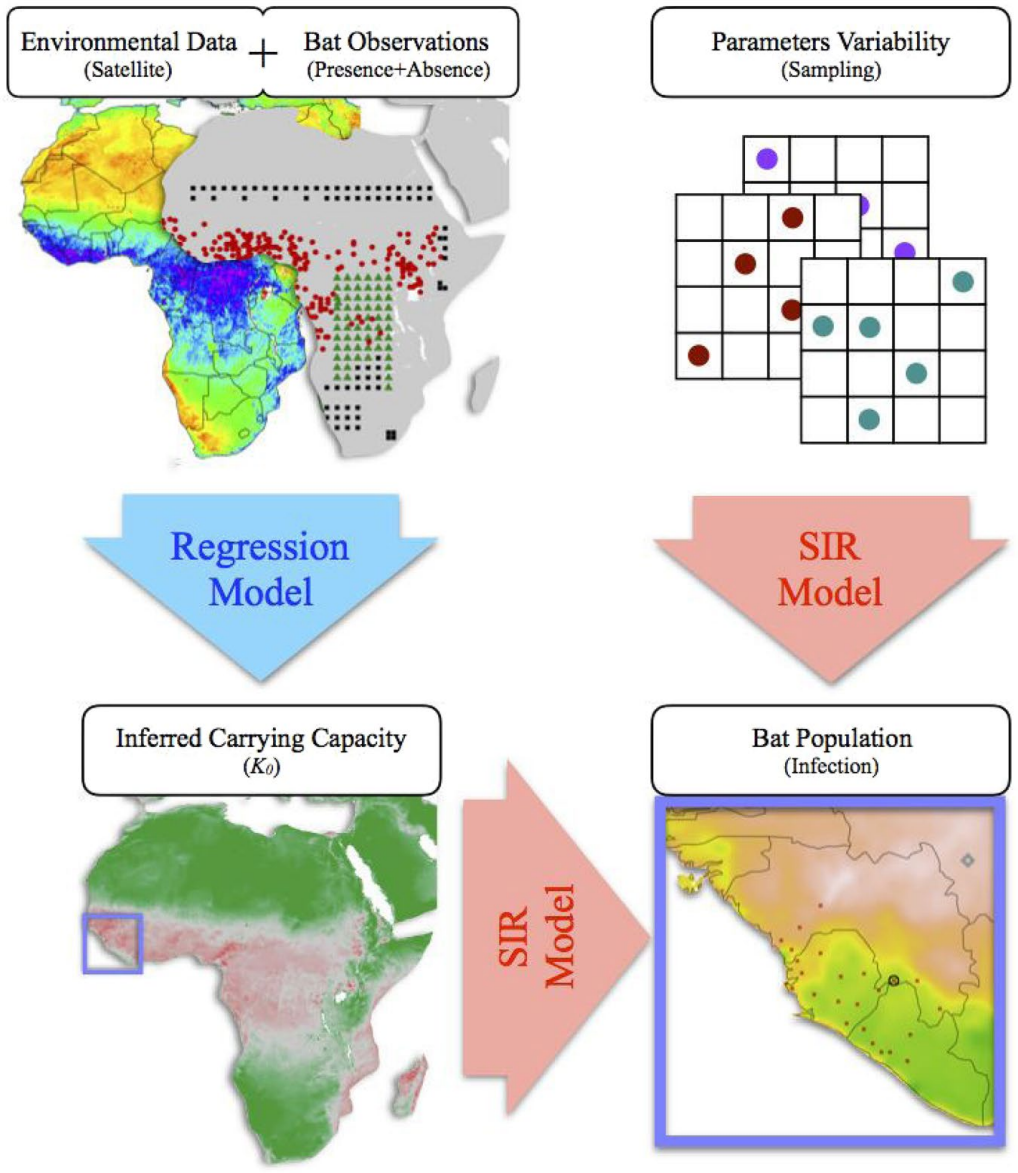
Framework flowchart. Collected satellite data of representative environmental factors for chiroptera ecology and presence-absence information about bat colonies are fed into the regression model to infer the spatial distribution of the bare carrying capacity, *K*_0_. The *K*_0_ map, together with a random set of sampled parameters, feeds a compartmental reaction-diffusion model for the bat population. The results of simulations, including the estimated basic reproductive number (see Results) and the density of bats in each state, are averaged over 512 random sets to generate a prediction. Map images were generated using R software packages “maps”, “gdal” and “raster”^55–57^.

The lack of sufficient data about several parameters involved in the compartmental model makes it difficult to obtain an accurate calibration and hence it diminishes the predictive capabilities of our framework. For example, available data about the transmission rate *e* among bats for EBOV is very limited, therefore we assumed that this rate is comparable to the infection rate of bats infected with Marburg. We estimated *e* observing seasonal variation of the bat seropositivity as given in^42^. Here we address the uncertainty about the characteristic rates by means of a sampling approach of the parameter space. Thus, we generate random sets of 512 instances for each parameter, assuming that each of them satisfies a uniform distribution within bounds as suggested by data and/or physical constraints. The list of sampled parameters and their estimated minimum and maximum values are given in Table 2. The sampling method we utilize is Latin hypercube sampling (LHS)^60^. We average the results of our simulations using the 512 sampling instances (i.e. sets). Assuming normality for the distribution of the mean value of the bat population, this sample size provides a ±2.0% accuracy on such mean with 95% confidence level. These confidence bounds were assessed based on the results of the proposed regression model^61^. The general flowchart of our framework is schematically presented in Fig. 5.

**Table 2:** Sampled parameters and range of variability.

Name	Parameter	Unit	Min	Max
Birth rate^62^	${b}_{{K}^{\ast }}$	$\frac{1}{Day}$	1.37*e*^−3^	4.11*e*^−3^
Death rate^62^	*c*	$\frac{1}{Day}$	0.68*e*^−4^	1.37*e*^−4^
Recovery rate^42^	*d*	$\frac{1}{Day}$	1.35*e*^−2^	4.10*e*^−2^
Infection rate^42,52^	*e*	$\frac{k{m}^{2}}{Bats\cdot Day}$	1.30*e*^−4^	6.50*e*^−4^
Curing rate^42^	*f*	$\frac{1}{Day}$	1.00*e*^−2^	5.00*e*^−2^
Diffusion rate^14,15^	${D}_{{K}^{\ast }}$	$\frac{k{m}^{2}}{Day}$	0	700
Env. Consumption rate^14^	*r*	$\frac{1}{Day}$	0	0.33
Bats Consumption rate^14^	*γ*	$\frac{1}{Day}$	0	0.26

## Electronic supplementary material

Movie S1

Movie S2

Movie S3

Supplementary Material
