# Supplementary material for: A Predictive Spatial Distribution Framework for Filovirus-Infected Bats
Source: Sci Rep. 2018 May 22;8:7970. doi: 10.1038/s41598-018-26074-4 (PMC5964142; doi:10.1038/s41598-018-26074-4)
Supplement: Supplementary file 4 — Supplementary Material [file 41598_2018_26074_MOESM4_ESM.pdf]

A Predictive Spatial Distribution Framework for Filovirus-Infected Fruit Bats

Graziano Fiorillo, Paolo Bocchini, and Javier Buceta

Supplementary Material

- Movie S1: Map of the mean inferred carrying capacity in West Africa (2014 outbreak region) during a period of one year. Map images were generated using R software packages “maps”, “gdal” and “raster” [1–3].

- Movie S2: Predicted evolution (one year) of the mean density of EBOV infected bats in the 2014 outbreak region. Map images were generated using R software packages “maps”, “gdal” and “raster” [1–3].

- Movie S3: Predicted evolution (one year) of the mean density of total bats (infected, susceptible, and recovered) in the 2014 outbreak region. Map images were generated using R software packages “maps”, “gdal” and “raster” [1–3].

- Simulation code and data files, http://doi.org/10.6084/m9.figshare.5633425

References

1. R Core Team. *R: A Language and Environment for Statistical Computing.* R Foundation for Statistical Computing, Vienna, Austria, 2013.

2. Roger Bivand, Tim Keitt, and Barry Rowlingson. *rgdal: Bindings for the Geospatial Data Abstraction Library*, 2016. R package version 1.1-10.

3. Robert J. Hijmans. *raster: Geographic Data Analysis and Modeling*, 2016. R package version 2.5-8.
